# Automated neonatal nnU-Net brain MRI extractor trained on a large multi-institutional dataset

**DOI:** 10.1038/s41598-024-54436-8

**Published:** 2024-02-26

**Authors:** Joshua V. Chen, Yi Li, Felicia Tang, Gunvant Chaudhari, Christopher Lew, Amanda Lee, Andreas M. Rauschecker, Aden P. Haskell-Mendoza, Yvonne W. Wu, Evan Calabrese

**Affiliations:** 1 Department of Radiology, University of California San Francisco, San Francisco, CA USA; 2 Division of Neuroradiology, Department of Radiology, Duke University Medical Center, Durham, NC 27710 USA; 3 Duke University School of Medicine, Durham, NC USA; 4 University of California San Francisco Weill Institute for Neurosciences, San Francisco, CA USA; 5 Duke Center for Artificial Intelligence in Radiology (DAIR), Durham, NC USA

**Keywords:** Medical research, Computer science, Magnetic resonance imaging, Paediatrics

## Abstract

Brain extraction, or skull-stripping, is an essential data preprocessing step for machine learning approaches to brain MRI analysis. Currently, there are limited extraction algorithms for the neonatal brain. We aim to adapt an established deep learning algorithm for the automatic segmentation of neonatal brains from MRI, trained on a large multi-institutional dataset for improved generalizability across image acquisition parameters. Our model, ANUBEX (automated neonatal nnU-Net brain MRI extractor), was designed using nnU-Net and was trained on a subset of participants (N = 433) enrolled in the High-dose Erythropoietin for Asphyxia and Encephalopathy (HEAL) study. We compared the performance of our model to five publicly available models (BET, BSE, CABINET, iBEATv2, ROBEX) across conventional and machine learning methods, tested on two public datasets (NIH and dHCP). We found that our model had a significantly higher Dice score on the aggregate of both data sets and comparable or significantly higher Dice scores on the NIH (low-resolution) and dHCP (high-resolution) datasets independently. ANUBEX performs similarly when trained on sequence-agnostic or motion-degraded MRI, but slightly worse on preterm brains. In conclusion, we created an automatic deep learning-based neonatal brain extraction algorithm that demonstrates accurate performance with both high- and low-resolution MRIs with fast computation time.

## Introduction

Magnetic Resonance Imaging (MRI) allows for the acquisition of high-resolution images with exceptional soft tissue contrast^1^, making it especially useful for evaluation of the brain, where it often informs patient medical management. For neonates, brain MRI is particularly important for assessment of patients with neonatal encephalopathy, where both the presence and pattern of brain injury can assist prognostication and treatment planning^2–7^. Advances in artificial intelligence (AI) and machine learning (ML) have allowed accurate prediction of functional outcomes in infants using MRI data^8–11^ taking advantage of the imaging information beyond what is reasonably utilized by human visual inspection alone. Image preprocessing is an essential step in standardizing data inputs for AI/ML algorithms, and ensures faster, more robust data processing while minimizing potential confounding features^12–18^.

Brain extraction, otherwise known as skull-stripping, is an essential step for virtually all AI/ML approaches to brain MRI analysis. While this process is well-established in adult brain models, there are limited extraction algorithms available for the neonatal brain. Brain extraction refers to the process by which brain tissue is segmented, and non-brain tissue, including the skull and extracranial soft tissues, is removed^12,14,16,18,19^. Brain extraction facilitates data de-identification by removing three-dimensional face data, which mitigates bias by preventing AI/ML algorithms from focusing on extracranial and facial soft tissues. Accurate automated brain extraction tools are important for improving standardization of the skull-stripping step, as manual editing is prone to variability, is time-consuming, and could influence the accuracy of associated AI/ML models. Historically, automated brain extraction tools have been based on thresholding and binary morphological operations, shape analysis, and/or atlas registration techniques^20–28^; however, the most modern and accurate approaches are based on deep learning (DL) with convolutional neural networks (CNNs)^29^. Despite recent progress with ML^16,29^, there is still a need for improved MRI brain extraction tools designed specifically for neonatal brains^30^, which differ from adult brains based on differences in morphology, signal contrast, and the increased frequency of motion artifact^13,15,17,18,24,29,31^.

DL-based brain extraction algorithm performance relies heavily on its training data, and generalizability can be limited by small training set sizes and lack of training data heterogeneity. Though models may learn to perform well on institution specific data, there is a need for more generalizable algorithms that can perform well on MRI data with varying acquisition parameters, field strength, and vendor platforms. To address this need for generalizability, we present ANUBEX (automated neonatal nnU-Net brain MRI extractor), a publicly-available DL-based algorithm for neonatal brain extraction based on the domain-leading nnU-Net architecture and trained on a large multi-institution dataset. We compare the performance of our algorithm to five publicly available algorithms spanning conventional, machine learning, and deep learning methods using a multi-institution external dataset^20,21,32,33^.

## Methods

### Study population

**Figure 1:**
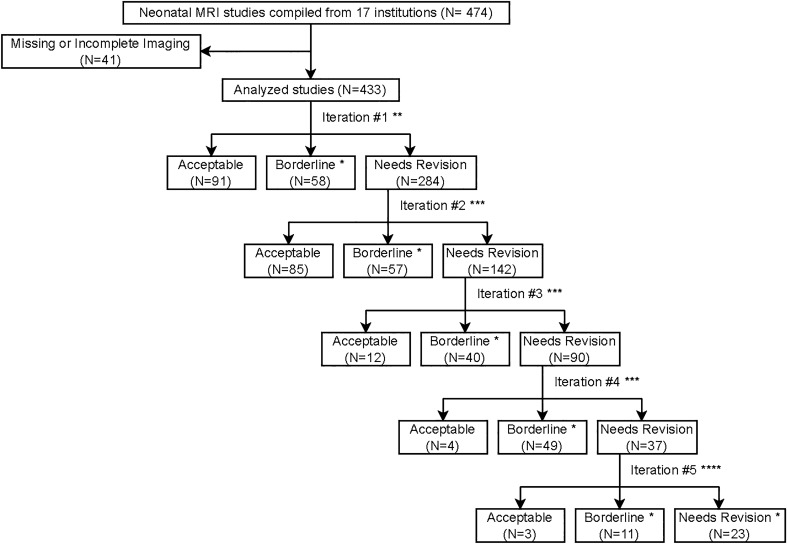
Flowchart describing the iterative brain masking process. * Studies were manually corrected. ** Iteration 1 used BET from FSL to generate automated brain masks. *** Iterations 2–4 used nnU-Net models to generate automated brain masks. Studies categorized as “borderline” were manually corrected. The nnU-Net models were subsequently retrained on the “acceptable” and newly corrected “borderline” studies, and new automated masks were regenerated for the “needs revision” studies. **** For iteration 5, all “borderline” and “needs revision” studies were manually corrected.

This was an Institutional Review Board approved ancillary study of the High-dose Erythropoietin for Asphyxia and Encephalopathy (HEAL) study^34–36^, which prospectively enrolled 501 neonates from 17 different institutions across the United States of America with moderate to severe encephalopathy at birth. Informed consent was previously obtained from all subjects and/or their legal guardian, and all methods were carried out in accordance with relevant guidelines and regulations. A subset of HEAL participants (N = 474) underwent neonatal MRI. Exclusion criteria included missing, incomplete, or severely artifact degraded T1-weighted MR imaging data (N = 41) resulting in a final study population of 433 participants from 17 different institutions (Fig. 1).

### Study data

Imaging data used for this study consisted of T1-weighted, T2-weighted, and diffusion-weighted imaging of the brain acquired as part of the HEAL trial. Scan parameters varied based on the imaging site and scanner platform. T1-weighted images included both three-dimensional gradient echo and two-dimensional spin echo imaging. T2-weighted images were two-dimensional Fast Spin Echo (FSE) imaging and diffusion-weighted images were Echoplanar Imaging (EPI). Other than in-plane resolution and slice thickness, scan parameters were not collected as part of the HEAL trial and are not consistently available for these data.

### Iterative deep learning model development

The ANUBEX architecture was designed using nnU-Net^37^, a self-configuring segmentation framework based on the popular U-Net architecture^38^, which is both widely used and has demonstrated domain leading segmentation performance on related tasks. Model training was accomplished using an iterative, human-in-the-loop AI approach. First, baseline automated brain masks were generated from T1-weighted images using a widely used tool for adult MRI brain extraction^21^. Next, all brain masks were manually reviewed by a single medical trainee (author JC) using ITK-SNAP^39^ and categorized as either “Acceptable,” “Borderline,” or “Needs Revision” using the following criteria:

#### Acceptable

Very little or no non-brain tissue included or brain tissue excluded; manual revision not expected to improve algorithm performance.

#### Borderline

Small amount of non-brain tissue included or brain tissue excluded; uncertain if manual revision will change algorithm performance.

#### Needs revision

Significant amount of non-brain tissue included or brain tissue excluded; manual revision expected to improve algorithm performance.

Studies labeled as “Borderline” were manually edited in ITK-SNAP by the same medical trainee. Next, all “Acceptable” and revised “Borderline” studies were used to train an instance of nnU-Net (single fold, random 80%/20% train/validation split). This model was then used to re-generate automated masks for the remaining “Needs revision” cases and the process was repeated for a total of five iterations, with each training instance reusing all previously labeled “Acceptable” and manually revised “Borderline” images. After five iterations, all remaining “Borderline” (N = 11) and “Needs revision” (N = 23) masks were manually edited to complete the training dataset.

Final model training using all the manually reviewed/corrected data (N = 433) was performed using a five-fold cross-validation approach with a standard random 80%/20% train/validation split for each fold. Model training was accomplished using a desktop computer equipped with two Nvidia RTX A600 40 GB graphics processing units running in parallel (one training fold per GPU). We developed two models, one trained on only T1-weighted imaging referred to as ANUBEX, and one trained on all three included sequences in a randomized manner referred to as ANUBEX Sequence Agnostic (ANUBEX-SA).

### External validation

Performance of the fully trained ANUBEX model was evaluated using an out-of-sample, external test set consisting of N = 39 T1-weighted images from two different sources: N = 20 from the developing Human Connectome Project (dHCP)^40^ consisting of high-resolution three-dimensional gradient echo T1-weighted imaging, and N = 19 from the NIH Pediatric MRI study^41^ consisting predominantly of lower resolution two-dimensional spin echo T1-weighted imaging. Corresponding T2-weighted images were also obtained from the dHCP test set. A single reviewer (author JC) manually reviewed the test set and manually generated each mask, which were subsequently used as ground truth for assessing automated brain masks. The proposed model was applied to the external test set using an ensemble of all five training folds.

Model performance was compared to five different publicly available automated brain extraction methods: BET, BSE, CABINET, iBEATv2, and ROBEX^20–22,32,33^. Each algorithm was applied to the external test set using default parameters. These benchmark comparison methods were chosen based on the following criteria: (1) publicly available, (2) out-of-the-box functionality (i.e. single command that runs on native data), and (3) based on a variety of different methods (e.g. shape analysis, atlas registration, deep learning).

### Sub-analyses

**Figure 2:**
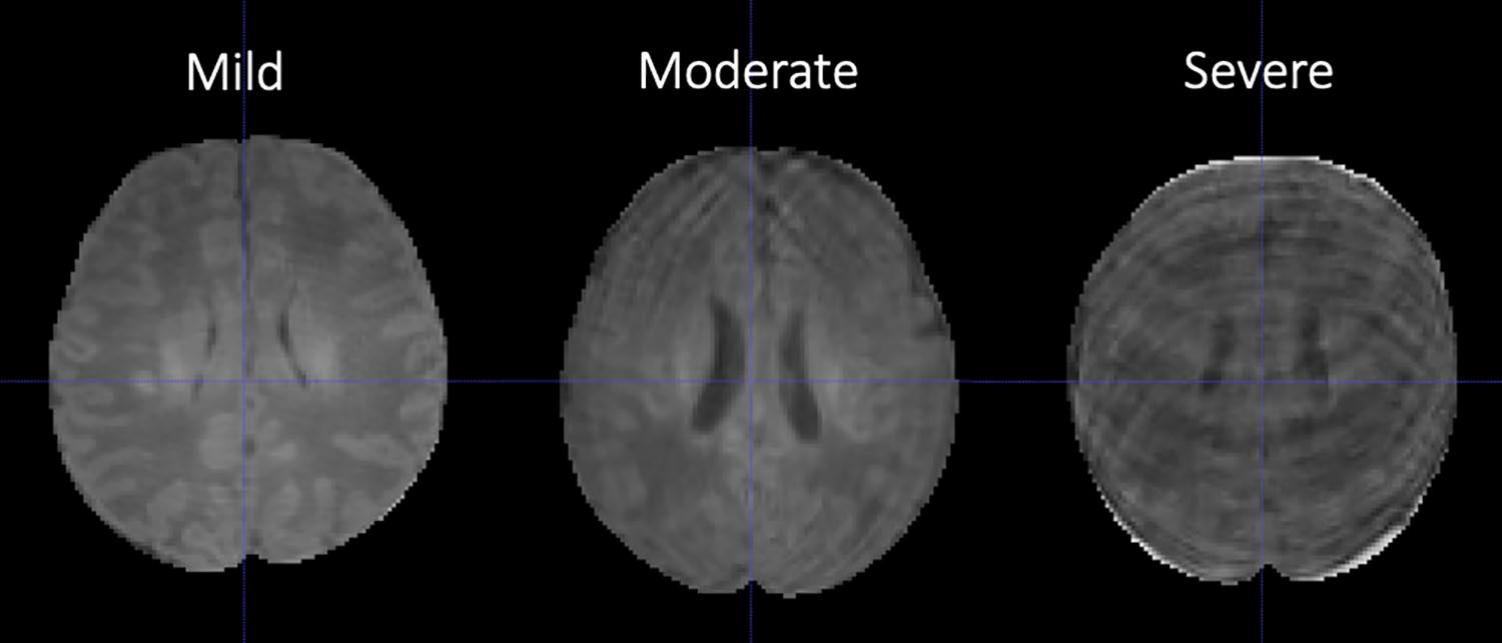
Examples of brain MRIs representing mild, moderate, and severe motion artifact.

In addition to the primary external validation described in the previous section, we performed several sub-analyses to evaluate model performance in different scenarios including different MRI sequences, preterm brain MRIs, and motion degraded brain MRIs. To address performance on different MRI sequences we evaluated ANUBEX-SA on T2-weighted imaging from the dHCP test set only, as the NIH data does not consistently contain T2-weighted imaging. To address performance on preterm brain MRIs, we evaluated ANUBEX on 18 T1-weighted brain MRIs performed before 36 weeks that were available in the dHCP dataset. To address performance in the setting of motion artifact, we evaluated the performance of ANUBEX on motion degraded validation data from the fivefold cross-validation. We chose this approach because there were insufficient exams with motion artifact in the testing data for a meaningful analysis. We identified 92/433 (21%) exams with at least moderate motion artifact and 341/433 (79%) exams with either mild or no significant motion artifact using the following objective criteria (Fig. 2):

#### Mild motion artifact

Slight motion artifact that does not obscure grey-white matter junction.

#### Moderate motion artifact

Motion artifact that incompletely obscures grey-white matter junction.

#### Severe motion artifact

Obvious motion artifact that completely obscures grey-white matter junction.

### Evaluation metrics and statistical analyses

The Dice coefficient was chosen as the primary metric for comparing manual and automated brain masks. The Dice coefficient compares the degree of spatial overlap between two binary images, ranging between 0 (no overlap) to 1 (perfect agreement), and is calculated as: Dice coefficient (A,B) = 2(A ∩ B)/(A + B) where (A ∩ B) is the union of masks A and B. Secondary metrics included sensitivity and specificity, calculated as Sensitivity = TP/(TP + FN), and Specificity = TN/(FP + TN) where TP is the number of true positive voxels in the mask, TN the number of true negative voxels, FP the number of false positive voxels, and FN the number of false negative voxels. Dice coefficients were calculated using custom Python code, and statistical comparisons between average Dice scores were computed using a two-sample, two-tailed t-test with a significance threshold of *p* < 0.05. We controlled for multiple comparisons using the Benjamini and Hochberg False Discovery Rate correction method.

### Ethical approval

This study was approved by the University of California, San Francisco Institutional Review Board as an ancillary study of the High-dose Erythropoietin for Asphyxia and Encephalopathy (HEAL) study.

## Results

### Study data and patient demographics

The final training dataset included N = 433 neonatal MRI studies from 17 institutions, 44% of which were female. The median gestational age (GA) at birth was 39.3 weeks (interquartile range [IQR] 38.1–40.3), with MRIs obtained between 96 and 144 h after birth^36^. The final external testing dataset included N = 39 neonatal MRI studies from two institutions, N = 20 from the dHCP and N = 19 from the NIH. The dHCP preterm sub-analysis data set included N = 18 MRIs. The median GA at scan of patients from the NIH, dHCP, and dHCP Preterm data sets, respectively, were 42.3 weeks (IQR 42.1–43.1), 40.6 weeks (IQR 39.7–40.9), and 34.5 weeks (IQR 34.0–35.3). The demographics of the NIH, dHCP, and dHCP Preterm data sets, respectively, were 53%, 30%, and 44% female. Basic participant demographic data is shown in Table 1. MRI resolution is shown in Table 2.

**Table 1:** Patient demographic information for the training and testing datasets.

	Training data set (N = 433) *	NIH data set (N = 19)	dHCP data set (N = 20) **	dHCP preterm data set (N = 18) **
Gestational Age at MRI (weeks) [Median (IQR)]	39.28 (38.14–40.28)	42.07 (42.07–43.14)	40.57 (39.68 –40.90)	34.50 (34.00–35.29)
Sex				
Female	191 (44.1%)	10 (52.6%)	6 (30.0%)	8 (44.4%)
Male	242 (55.9%)	9 (47.4%)	14 (70.0%)	10 (55.6%)
Self-reported Race of Maternal Parent				
White	308 (71.1%)	12 (63.2%)		
Black	56 (12.9%)	0 (0%)		
Asian	29 (6.7%)	1 (5.3%)		
Other	40 (9.2%)	6 (31.6%)		
Self-reported Ethnicity of Maternal Parent				
Hispanic	113 (35.3%)	0		
Non-Hispanic	320 (73.9%)	19 (100%)		

*Training Data Set from the HEAL Study reported only Gestational Age at Birth. Scans were acquired generally 4–6 days after birth.

**dHCP and dHCP Preterm Data Sets do not contain Race/Ethnicity information.

**Table 2:** Slice resolution for N = 433 T1-weighted MRIs. Resolution Z-axis represents slice thickness.

	Resolution X-axis	Resolution Y-axis	Resolution Z-axis
Range (mm)	0.60–1.20	0.39–1.07	0.39–5.2
Mean (mm)	0.98	0.97	1.06
Median (mm)	1	1	1

### Model training

Final model training lasted approximately 36 h. Training and validation loss (Dice) decreased appropriately throughout the training process. Final trained model weights are freely available online (https://github.com/ecalabr/nnUNet_models).

### External validation and performance evaluation

**Figure 3:**
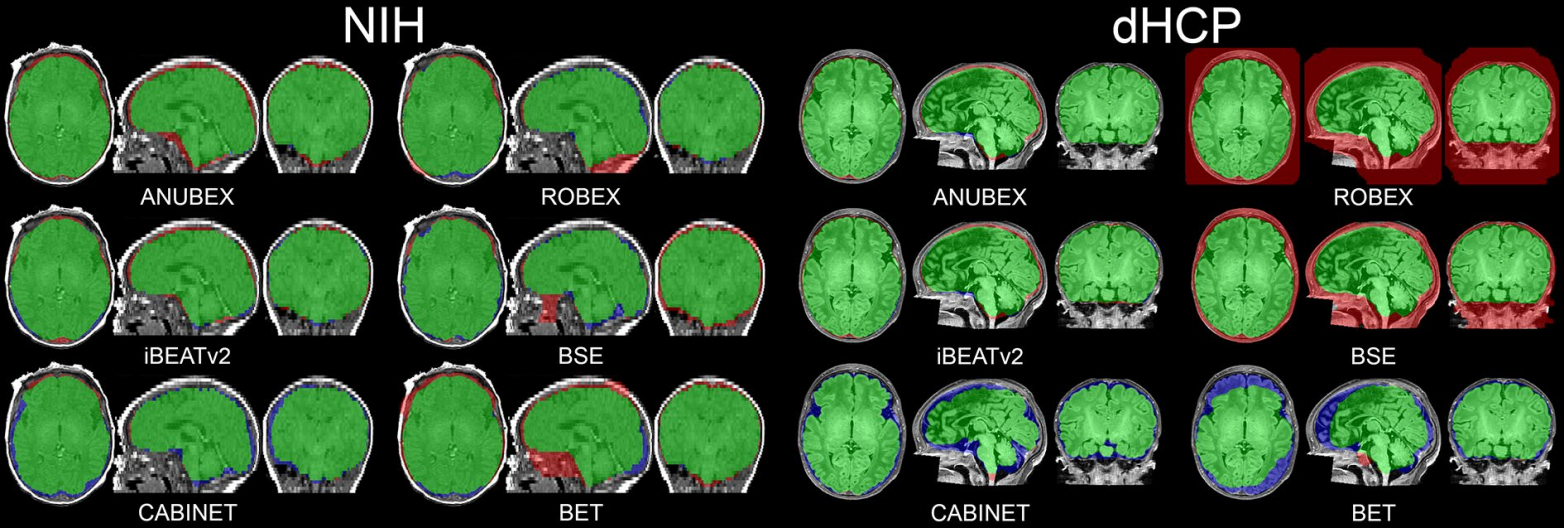
Comparison of masks generated by 6 automatic brain segmentation tools on 2 randomly selected MRIs, one from the NIH dataset (left two columns) and one from the dHCP dataset (right two columns). Green pixels represent mask pixels that appropriately capture true brain as determined by gold standard manual segmentation. Red pixels represent mask pixels that capture nonbrain pixels. Blue pixels represent true brain that was not captured by mask pixels.

**Figure 4:**
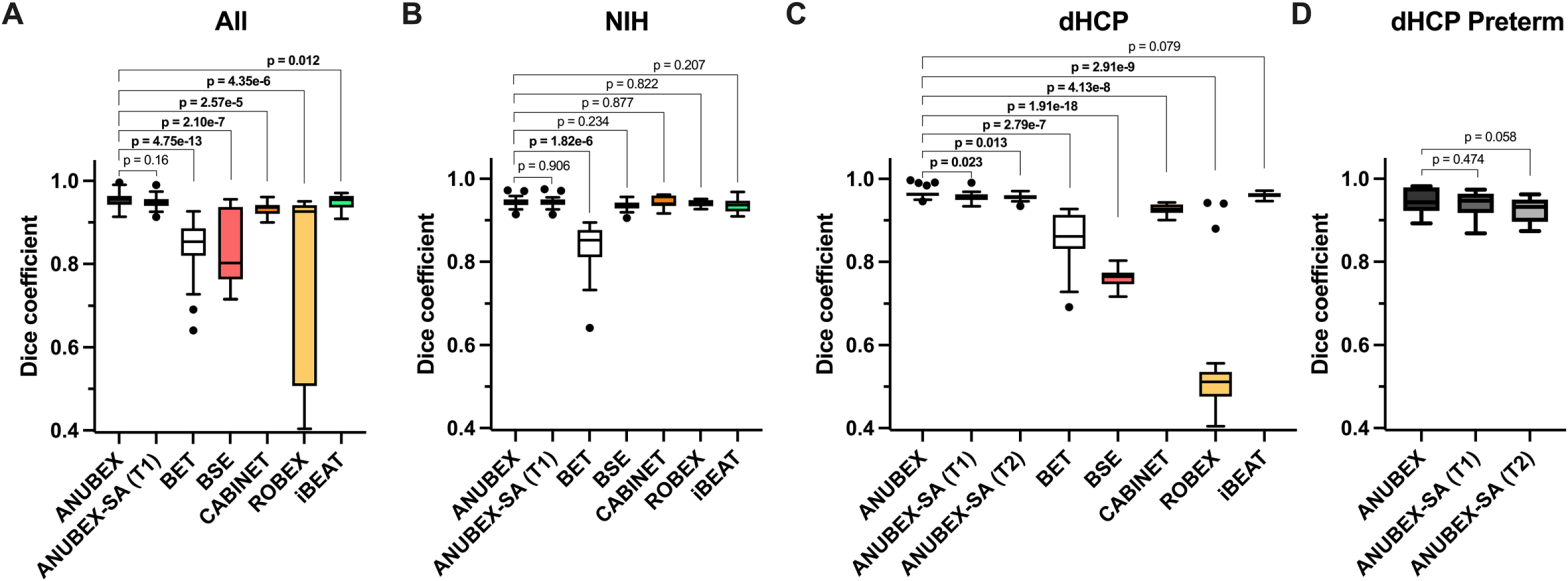
Box and whisker plots of Dice similarity coefficients across 6 unique automatic brain segmentation tools and 1 application of our model (ANUBEX-SA) for the (**A**) All, (**B**) NIH, (**C**) dHCP, and (**D**) dHCP Preterm datasets (refer to Table 3 for tabulated values). Paired two-tail T-tests were performed between ANUBEX and each comparator, with the Benjamini and Hochberg False Discovery Rate correction method applied to p-values to control for multiple comparisons.

External validation and performance evaluation were performed using the multi-institution external test dataset (N = 39). Processing time for all 39 studies in the external test set took 330.34 s or an average of 8.5 s per study using an Nvidia RTX A6000 GPU. Results from ANUBEX were compared to results from 5 other publicly available brain extraction tools: BET, BSE, CABINET, iBEATv2, and ROBEX^20–22,32,33^. Dice scores for all models evaluated on the testing dataset are provided in Table 3. Example brain masks generated by each algorithm are shown in Fig. 3. The Dice coefficient of our model was the highest of all methods tested with a mean ± standard deviation of 0.955 ± 0.017 (Fig. 4A). The next best performing model (iBEATv2) yielded an average Dice of 0.949 ± 0.017, followed by CABINET at 0.934 ± 0.015. Other evaluated methods yielded average Dice scores below 0.85. Our model showed a small but statistically significant improvement in performance compared to the two other deep learning algorithms CABINET (*p* < 0.001) and iBEATv2 (*p* = 0.012) and a larger statistically significant difference between the non-deep learning algorithms ROBEX, BSE, and BET. Sub-analysis of algorithm performance on the external test set by site revealed a trend towards better performance on the dHCP (3D) image data (Fig. 4C) compared to the NIH (2D) data (Fig. 4B). Notably, our algorithm showed the highest performance of all algorithms tested for both dHCP and NIH data.

**Table 3:** Model performance metrics are presented for each of the test sets.

	Dice coefficient	*p*-value ^a^	Sensitivity	Specificity	PPV
All^b^					
ANUBEX	0.955 ± 0.017		0.932	0.996	0.982
ANUBEX-SA (T1)	0.950 ± 0.014	0.160	0.926	0.995	0.977
BET	0.845 ± 0.063	4.753 × 10^–13^ *	0.856	0.973	0.876
BSE	0.845 ± 0.090	2.101 × 10^–7^ *	0.774	0.997	0.963
CABINET	0.934 ± 0.015	2.572 × 10^–5^ *	0.988	0.981	0.887
ROBEX	0.746 ± 0.220	4.350 × 10^–6^ *	0.680	0.996	0.960
iBEAT v2	0.949 ± 0.017	0.012 *	0.916	0.999	0.986
NIH					
ANUBEX	0.944 ± 0.014		0.895	1.000	0.999
ANUBEX-SA (T1)	0.943 ± 0.014	0.9057	0.895	1.000	0.998
BET	0.833 ± 0.063	1.819 × 10^–6^ *	0.723	0.999	0.991
BSE	0.935 ± 0.011	0.234	0.943	0.994	0.929
CABINET	0.942 ± 0.015	0.877	0.979	0.993	0.909
ROBEX	0.941 ± 0.008	0.822	0.953	0.995	0.931
iBEAT v2	0.937 ± 0.018	0.207	0.895	0.999	0.985
dHCP					
ANUBEX	0.966 ± 0.014		0.967	0.992	0.966
ANUBEX-SA (T1)	0.956 ± 0.012	0.023 *	0.955	0.990	0.957
ANUBEX-SA (T2)	0.956 ± 0.008	0.013 *	0.937	0.994	0.976
BET	0.857 ± 0.065	2.788 × 10^–7^ *	0.982	0.948	0.766
BSE	0.759 ± 0.022	1.914 × 10^–18^ *	0.614	0.999	0.995
CABINET	0.927 ± 0.012	4.130 × 10^–8^ *	0.996	0.969	0.866
ROBEX	0.561 ± 0.159	2.914 × 10^–9^ *	0.422	0.998	0.988
iBEAT v2	0.961 ± 0.006	0.079	0.937	0.997	0.987
dHCP Preterm					
ANUBEX	0.947 ± 0.030		0.924	0.996	0.972
ANUBEX-SA (T1)	0.940 ± 0.028	0.474	0.910	0.996	0.972
ANUBEX-SA (T2)	0.925 ± 0.028	0.058	0.867	0.999	0.992

*False Discovery Rate corrected *p*-value < 0.05.

^a^*p*-value was calculated with a paired two-tailed t-test between Dice scores of the ANUBEX model and the comparison model with Benjamini and Hochberg False Discovery Rate *p*-value correction.

^*b*^Aggregate external test set included both NIH and dHCP data sets but not preterm data. ANUBEX-SA (T1) refers to the sequence agnostic model trained on T1-, T2-, and diffusion-weighted images and evaluated on T1-weighted images, and ANUBEX-SA (T2) indicates that this model was evaluated on T2-weighted images.

### Sub-analyses

Sub-analysis results are presented in Table 3 and Fig. 4. ANUBEX-SA (trained on T1-, T2-, and diffusion-weighted images) showed similarly high performance on T1-weighted imaging from both test sets (average Dice = 0.956 ± 0.012 for dHCP and Dice = 0.943 ± 0.014 for NIH) and performance on T2-weighted imaging from the dHCP test set was nearly identical (average Dice = 0.956 ± 0.008). We detected small but statistically significant decreases in performance of ANUBEX-SA compared to ANUBEX for the dHCP test set but not for the NIH test set or aggregate test set.

ANUBEX performance on the 18 preterm (< 36 weeks gestational age) brain MRIs from the dHCP yielded an average Dice = 0.947 ± 0.030, which was slightly worse compared to performance on term dHCP MRI data (*p* = 0.015). ANUBEX-SA performance was average Dice = 0.940 ± 0.028 for T1-weighted images and 0.925 ± 0.028 for T2-weighted images, which was not significantly different compared to regular ANUBEX performance on preterm T1-weighted images (Fig. 4D).

ANUBEX performance in the setting of moderate or severe motion artifact was evaluated on validation data from the fivefold cross-validation, which results in elevated Dice scores compared to test set data but still allows comparison of performance between MRIs with and without motion artifact. Average validation Dice score for ANUBEX was 0.986 ± 0.021 for the group with at least moderate motion artifact compared to 0.988 ± 0.020 in the group without significant motion artifact. This difference was not statistically significant (*p* = 0.470).

## Discussion

In this study, we evaluated ANUBEX, a new deep learning-based model for neonatal MRI brain extraction based on the widely used nnU-Net architecture. Model performance was evaluated on an independent, multi-institution, external dataset and results were compared to five other publicly available brain extraction methods including deep learning-based and non-deep learning-based methods: BET, BSE, CABINET, iBEATv2, and ROBEX. Compared to the other methods we evaluated, our model demonstrated superior brain extraction performance on both 2D and 3D neonatal brain MRIs. Specifically, there was a small but significant improvement in performance compared to the other two deep learning-based methods (CABINET and iBEATv2) and a larger significant difference compared to the non-deep learning-based methods. Based on sub-analysis results, our model performs slightly worse on brain MRIs of preterm infants as compared to term infants, an expected outcome given our model was trained on term and near-term infants. We did not find significant differences in performance between our T1-weighted model (ANUBEX) or our sequence agnostic model (ANUBEX-SA) whether evaluated on T1- or T2-weighted images, and model validation performance was not significantly different in moderately to severely motion degraded versus non to mildly motion degraded images.

Our approach to model generation has several potential advantages that may have contributed to the observed performance increase. First, we employed an iterative semi-automated approach to ground truth brain mask generation, which allowed increased efficiency and consistency. Second, we utilized a multi-institutional dataset from the HEAL trial as training data for our deep learning algorithm in order to create a more generalizable model across different institutions. By training with a larger and more heterogeneous sample including variation in MRI manufacturer, model, software, and imaging parameters^36^, our model can potentially achieve higher accuracy in neonatal skull stripping across various institutions in comparison to studies performed with a smaller and institution specific dataset. For example, our model showed improved performance with both high-resolution (0.8 × 0.8 × 1.6 mm) 3D imaging (dHCP) and thicker slice (1.0 × 1.0 × 3.0 mm) 2D imaging (NIH), which is likely attributable to the training data heterogeneity. Comparatively, iBEATv2 was trained on only the high-resolution Baby Connectome Project dataset (resolution 0.8 × 0.8 × 0.8 mm), and ROBEX was trained on a proprietary dataset of 92 healthy adult subjects (downsampled to lower resolution 1.5 × 1.5 × 1.5 mm)^33^. Finally, our model was generated using the widely used nnU-Net architecture, which has “out-of-the-box” functionality and has shown domain-leading performance in other medical image segmentation tasks. The use of nnU-Net also allows straightforward sharing of trained model weights and can lower barriers to implementation and use in future research projects.

This study has several important limitations. First, the use of data from the HEAL trial limits the scope of brain pathology included in the training data. HEAL study participants all had moderate to severe encephalopathy and did not have other major structural brain abnormalities. While several other intracranial pathologies were present in HEAL participants (e.g., infarcts, hemorrhages, hydrocephalus) these were not rigorously documented nor was the model specifically tested for brain extraction performance in the setting of any brain abnormality. Therefore, performance in the setting of brain structural pathology may be degraded. Second, we focused exclusively on the early neonatal period (< 44 weeks GA at scan) and therefore performance in patients older than 44 weeks GA may be degraded. Finally, comparison with other publicly available models was not exhaustive as several previously published algorithms had webpages that were inactive or code that was non-functional on modern software stacks.

Because accurate brain tissue segmentation is key to subsequent image analysis and volumetric measurements, necessary future steps would include further evaluation of the accuracy of our model on patients outside of the neonatal age range, such as in young children or adults, and assessing our model’s utility on brains with diverse structural pathology. We were not able to uniformly perform sub-analyses on all other algorithms because of varying abilities to support T2-weighted imaging.

In conclusion, we propose an application of nnU-Net to create a newer high-accuracy automatic neonatal brain extraction algorithm trained on a large multi-institutional dataset to improve generalizability across MRI acquisition parameters. Our model demonstrates accurate performance with both high- and low-resolution MRIs and is designed to have a lower barrier to use as an “out-of-the-box” ready software with fast computational time.

## Data Availability

Trained model weights are available through the corresponding author or online at: https://github.com/ecalabr/nnUNet_models

## Abbreviations

JVC: Study design, literature search, data acquisition or analysis, manuscript drafting, manuscript figures/tables, manuscript revision
YL: Study design, data acquisition or analysis, manuscript drafting, manuscript revision
FT: Data acquisition or analysis, manuscript drafting, manuscript figures/tables, manuscript revision
GC: Data acquisition or analysis, manuscript revision
CL: Manuscript revision
AL: Manuscript revision
AMR: Manuscript revision
APH: Manuscript figures/tables, manuscript revision
YWW: Data acquisition or analysis, manuscript revision
EC: Study design, literature search, data acquisition or analysis, manuscript drafting, manuscript figures/tables, manuscript revision
